# Three-Dimensional Cultured Human Dermal Papilla Cells in HGC-Coated Environments Enhance Hair Regeneration and Artificial Skin Integration

**DOI:** 10.34133/bmr.0018

**Published:** 2024-04-12

**Authors:** Thuy Trang Truong, Heejin Park, Kyoung Hwan Park, Jin Jung Song, Byoung-Seok Lee, Kang Moo Huh, Sun-Woong Kang

**Affiliations:** ^1^ Research Group for Biomimetic Advanced Technology, Korea Institute of Toxicology, Daejeon 34114, Republic of Korea.; ^2^ Department of Polymer Science and Engineering, Chungnam National University, Daejeon 34134, Republic of Korea.; ^3^ Department of Toxicological Evaluation and Research, Korea Institute of Toxicology, Daejeon 34114, Republic of Korea.; ^4^ Human and Environmental Toxicology School, University of Science and Technology, Daejeon 34114, Republic of Korea.

## Abstract

The rapid development of biomimetic materials in the field of regenerative medicine has made it possible to mimic natural cellular environments and allow in vitro systems to replace in vivo systems. In particular, the emergence of hexanoyl glycol chitosan (HGC) is playing an important role the development of 3-dimensional (3D) cell culture and tissue engineering. We employed HGC-coated dishes to cultivate human hair follicle dermal papilla (HDP) cells in 3D spheroids, assessing their ability to form hair-like structures. The study also tested the effect of minoxidil on these spheroids and explored their integration into artificial skin models. HDP cell spheroids successfully formed hair-like structures within the 3D culture. Minoxidil treatment showed enhanced hair growth in spheroids cocultured with keratinocytes. In addition, transplantation of these spheroids into artificial skin led to the formation of functional papilla structures, suggesting a closer mimicry of human skin. Hair-like structure formation and successful integration into artificial skin models pave the way for innovative approaches in hair loss treatment research, cosmetic, and pharmaceutical evaluations and skin restoration therapies.

## Introduction

The field of regenerative medicine is quickly growing, blending nature’s complexity with human innovation. Central to this convergence is the exploration and application of biomimetic materials [1–3]. These materials play a pivotal role in directing cellular behavior, bridging the divide between laboratory (in vitro) experiments and real-world biological systems (in vivo). They stand as potential harbingers for significant leaps in tissue engineering and therapeutics. Among the various innovative strategies in regenerative medicine, temperature-sensitive polymers have emerged as a key area of interest. Specifically, these polymers, with their unique ability to change phases in response to external stimuli, are finding applications in diverse areas like cell culture, drug delivery, and tissue engineering. One such notable polymer is hexanoyl glycol chitosan (HGC), a derivative of the naturally occurring biopolymer chitosan. The attention toward HGC is largely due to its dual features: temperature sensitivity combined with ultralow adhesion properties. These properties are potentially useful for cell culture and tissue engineering [4–6].

A growing area in this field is 3-dimensional (3D) cell culture. Traditional 2-dimensional cultures, while useful, often fail to replicate the complex microenvironments and cell–cell interactions inherent to living tissues. In contrast, 3D spheroid cultures promise a closer approximation to in vivo systems. They have shown a superior ability to emulate organ-specific functionalities and cellular behaviors, forming a more accurate bridge between the petri dish and the human body [7–10]. Such platforms are not only pivotal for therapeutic advancements but also offer alternative, and often more representative, research models that can potentially reduce or replace animal testing [1,8,11–14]. However, while the benefits of 3D cell cultures are undeniable, the creation of functional tissue constructs, especially artificial skin, presents a considerable challenge. Existing artificial skin models have limitations. They often lack the intricate microarchitecture of natural skin, resulting in suboptimal mechanical strength, permeability, and sensory feedback. Furthermore, while they can replicate some of the superficial functions of skin, they fall short in emulating complex physiological processes, such as hair growth, sweat production, and nuanced immune responses [15,16]. This gap underscores the imperative need for the development of highly functional artificial skin that not only serves as a protective barrier but also replicates the full suite of skin's multifaceted roles [17].

**Fig. 1.**
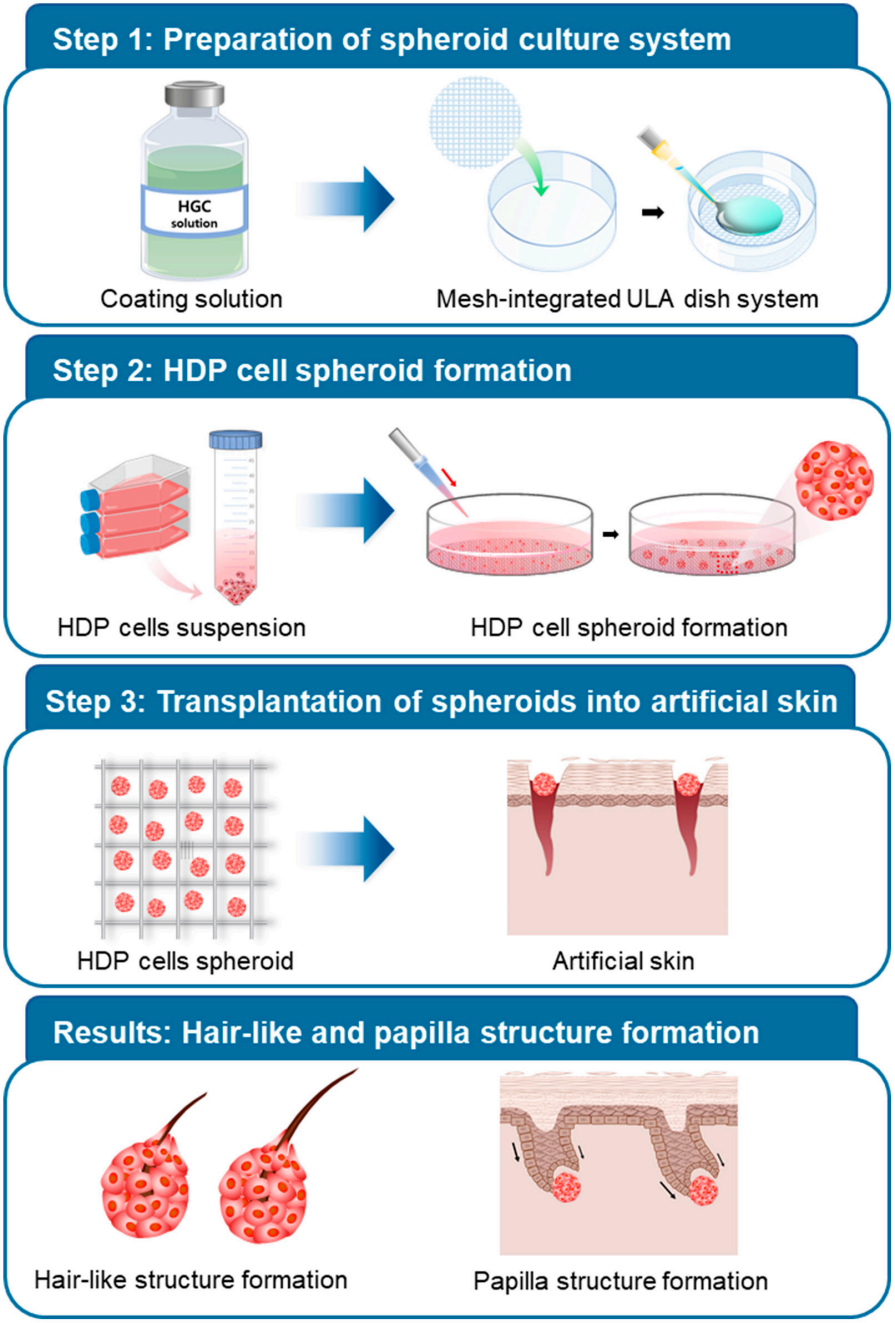
Schematic illustration for research process and results.

In the broader context of 3D cell cultures, a significant exploration is its application in treating hair growth disorders, such as alopecia. The dermal papilla, situated at the hair follicle’s base, is key to hair morphogenesis and cyclic growth. Understanding and potentially modulating these cells might be the key to hair regrowth therapies [14,15,18]. Here, the unique properties of HGC, particularly its ultralow adhesion, may be instrumental in fostering the cultivation of papilla cells, enhancing their capacity for hair formation. This investigation probes into the feasibility of integrating these bioengineered cell constructs within a strategically engineered artificial skin, designed to mimic the native papillary architecture of human skin (Fig. 1). The primary goal of this study is to elucidate the potential of HGC in 3D spheroid formation, emphasizing its implications in regenerative medicine, especially hair regeneration, and the quest for advanced research models.

## Materials and Methods

### Materials

Triple-distilled water, methanol, and acetone were obtained from Samchun Chemical (CheonAn, Korea). Petri dishes, normal cell culture dishes, and ultralow attachment (ULA) dishes were acquired from SPL (Pocheon, Korea), Thermo Fisher Scientific (MA, USA), and Corning (NY, USA), respectively. Human hair follicle dermal papilla (HDP) cells and culture medium were procured from CEFO (Seoul, Korea). Human adult primary epidermal keratinocytes (KCs) and their culture medium kit were obtained from American Type Culture Collection (ATCC) (VA, USA). A 4% v/v formaldehyde solution, Triton X-100, and DPX Mountant were sourced from Sigma-Aldrich. The Live/Dead Viability/Cytotoxicity Kit for mammalian cells was purchased from Invitrogen (CA, USA). Dulbecco’s phosphate-buffered saline (GIBCO) was obtained from Thermo Fisher Scientific. Minoxidil was procured from Sigma. EpiDerm Full Thickness 400 (EpiDermFT) was acquired from MatTek (MA, USA). Antibodies Ki67, Keratin 40 (K40), and Keratin 14 (K14) were purchased from Abcam. Trichohyalin (TCHH) and GATA3 were obtained from Invitrogen. Keratin 5 (K5), SOX9, and LEF1 were purchased from Cell Signaling Technology.

### Cell cultures

The HDP cells were passaged upon reaching 80% confluency, utilizing trypsin EDTA/Neutralizing Solution (ATCC PCS-999-003/ ATCC PCS-999-004, Manassas, VA, USA). All cells were cultured at 37 °C in a humidified atmosphere with 5% CO₂. The medium was changed every 2 d. Human adult primary epidermal keratinocytes were maintained in dermal cell basal medium (ATCC PCS-200-030, Manassas, VA, USA) supplemented with a keratinocyte growth kit (ATCC PCS-200-040, Manassas, VA, USA). The cells were passaged upon reaching 80% confluency using trypsin EDTA/Neutralizing Solution. The HDP cells and keratinocytes at a low passage number (3 to 4 passages) were used for this study.

### Cell proliferation assay

Cell proliferation was assessed using the cell counting kit-8 (CCK-8, Dojindo, Japan). HDP cells were seeded at densities of 2.0, 5.0, and 10.0 × 10^3^ cells/well in 96-well plates (Corning, NY, USA). Proliferation was analyzed over a period of 8 d. The absorbance of the samples was measured at an optical density of 450 nm, and the resulting data were used to plot the proliferation curves.

### Preparation of HGC-coated dish and HDP cell spheroids

For the preparation of HDP cell spheroids, we utilized a 35-mm dish specifically designed for spheroid formation [19,20]. Briefly, the mesh was precisely cut to a diameter of 35 mm, and a ring was used to secure the cut mesh to a 35-mm petri dish (SPL life science, Pocheon, Korea). For the HGC coating, HGC was solubilized in tertiary distilled water to achieve a 0.1 wt% concentration. This prepared HGC solution was uniformly distributed across petri dish integrated with mesh through careful pipetting, ensuring an even coating. Subsequently, these dishes were subjected to a preconditioning step at 37 °C for 1 h to facilitate prewetting. Following this phase, the solution was meticulously decanted from the dishes, which were then placed in an oven set at 55 °C and left to dry for a duration of 24 h. After that, it was stored at room temperature until used in cell culture experiments.

Prior to cell seeding, the dish underwent 3 sequential rinses using 70% ethyl alcohol and phosphate-buffered saline (PBS; Gibco, NY, USA), followed by the removal of all residual liquid. After that, HDP cells were plated onto mesh-integrated HGC-coated dishes, which were specifically designed to facilitate uniform spheroid formation. Different cell densities (2.0, 5.0, and 10.0 × 10^5^ cells/dish) were tested to examine the impact of cell concentration on the size of the spheroids. The spheroid formation cultures were maintained for 7 d. After this period, the cell density of 10.0 × 10^5^ cells/dish was selected for further experimentation. The spheroids were examined under a light microscope (Leica DMi8, Leica, Wetzlar, Germany) on days 1, 3, and 7.

For the coculture spheroids, a 1:1 mixture of HDP cells and keratinocytes was prepared in their respective media. This suspension was then plated into a mesh-integrated HGC-coated dish at a total density of 10.0 × 10^5^ cells/dish. All cultures were incubated at 37 °C with 5% CO₂, and the medium was changed every 2 d.

### Minoxidil treatment on HDP cell spheroids

Minoxidil was initially dissolved in ethanol and then diluted with cell basal medium to achieve a final concentration of 10 μM. The application of the minoxidil solution began on day 1 of the spheroid culture in order to minimize interference with the formation of the spheroids. After 1 d, the medium was removed and replaced with fresh medium containing a 10 μM concentration of minoxidil. One week later, each spheroid was individually transferred to separate wells of a 96-well plate to facilitate easy monitoring. Control specimens were maintained without minoxidil, but all other conditions were kept identical, including the addition of keratinocytes.

### Hair shaft formation within HDP cell spheroids

After 1 week of spheroid formation on mesh-integrated HGC-coated dishes, the HDP cell spheroids were transferred to ULA dishes (#3261, Corning, NY, USA) for extended culture. To confirm hair shaft growth within the spheroids, various techniques such as scanning electron microscopy (SEM), hematoxylin and eosin (H&E) staining, and immunostaining were employed. The primary antibodies used in this study included Ki67 (Abcam, ab15580, 1:200; Abcam, ab16667, 1:200), K40 [AE13 clone] (Abcam, ab16113, 1:100), TCHH [AE15 clone] (Invitrogen, MA5-18026, 1:50), K5 (Cell Signaling Technology, 71536, 1:50), K14 (Abcam, ab7800, 1:500), SOX9 (Cell Signaling Technology, 82630, 1:100), LEF1 (Cell Signaling Technology, 2230S, 1:100), and GATA3 (Invitrogen, MA1-028, 1:200).

### Incorporating HDP cell spheroids into full-thickness skin models

After a specific preparation protocol, EpiDerm Full-Thickness skin models (EpiDermFT, MatTek Corp., MA, USA) were subjected to a simulated wound using a 50-ml syringe needle, achieving a depth of 0.5 to 1 mm. Subsequently, HDP cell spheroids were introduced into this wound and positioned within the dermal fibroblast-collagen layer. The culture medium was gradually transitioned from EpiDermFT medium to HDP medium. Tissues without embedded HDP cell spheroids served as the control group. After 4 weeks of culture, the tissues were analyzed using H&E staining and immunohistochemistry.

### Histology and immunohistochemistry

Spheroids exhibiting hair shaft-like structure formation, along with EpiDermFT tissues, were harvested and fixed using 4% paraformaldehyde. These tissues were then embedded in paraffin blocks and sectioned into 4-μm slices. The slices underwent deparaffinization and antigen retrieval using a retrieval solution (DAKO, K8005). They were then permeabilized with PBS containing 0.5% Triton X-100 for 15 min. For H&E staining, the sections were stained with hematoxylin for 10 min and eosin for 1 min, followed by a dehydration process through a series of ethanol and xylene solutions before mounting with DPX Mountant. For immunofluorescence staining, the slices were incubated overnight with the previously mentioned primary antibodies, diluted in an antibody diluent containing background-reducing components (DAKO, S3022), at 4 °C. After 3 PBS washes, the slices were incubated with fluorescent secondary antibodies for 120 min at room temperature in the dark. The secondary antibodies used in this study were donkey anti-goat 488 (Invitrogen, A11055, 1:400) and donkey anti-rabbit 647 (Invitrogen, A31573, 1:400). Following 3 additional PBS washes, the slices were counterstained with 4',6-diamidino-2-phenylindole (DAPI) (Vecta Shield) for nuclear staining and then mounted on slides. The tissue sections were subsequently imaged using a fluorescence microscope (Nikon Eclipse TS2), a confocal microscope (Zeiss), and a Lionheart FX automated microscope (Biotek, USA).

### Image acquisition

Optical and immunofluorescence images were acquired using a Leica DMi8 microscope and an Olympus FluoView FV3000 inverted confocal microscope and were processed with ImageJ software. H&E images were captured using a GT 450 slide scanner (Leica).

### Statistical analysis

Statistical analysis was performed using 1-way analysis of variance followed by Tukey’s multiple comparison posttest. This analysis was conducted utilizing Origin 8 (OriginLab) and Prism 8 (GraphPad, CA, USA) software. Data are presented as mean ± standard deviation (SD).

## Results

### Preparation of HDP cell spheroids

We first increased the number of HDP cells using serial passaging process in 2-dimensional culture. Within 24 h after placing them in a standard cell culture dish, most of the cells attached to the bottom of the dish and spread out flat (Fig. S1A). Over time, they often took on a shape like fibroblasts, becoming elongated or thin. By 7 d, they almost completely covered the bottom of the dish. The cell growth rate was pretty steady, irrespective of the number of cells plated. The densest cultures consistently exhibited the highest cell count (Fig. S1B).

**Fig. 2.**
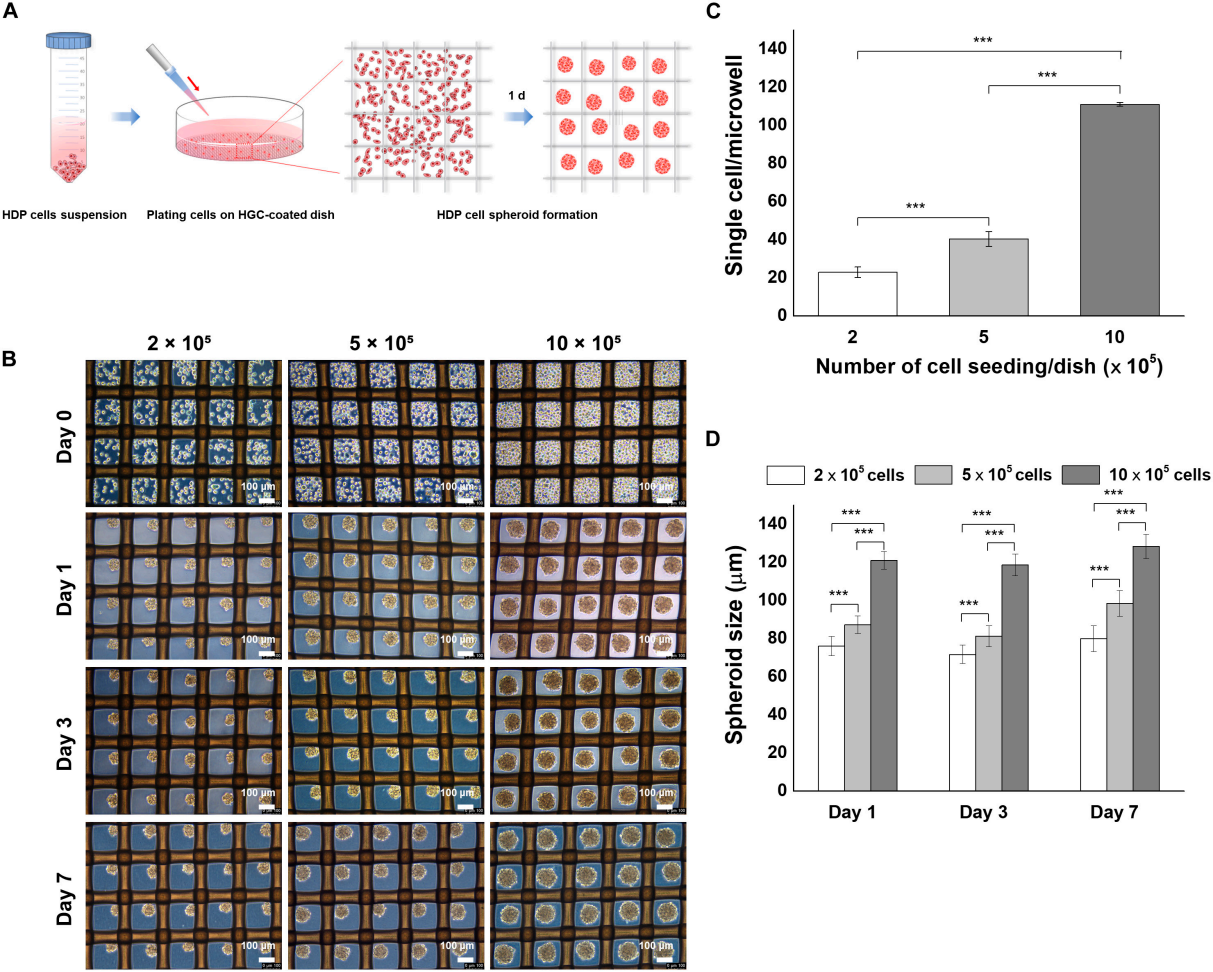
Spheroid formation of human dermal papilla (HDP) cells and regulation of spheroid size according to cell density in mesh-integrated HGC-coated dishes. (A) Schematic illustration of the process by which HDP cells form spheroids in mesh-integrated HGC-coated dish. (B) Phase-contrast images of HDP cell spheroids cultured for 7 d at different seeding densities (2.0 × 10^5^, 5.0 × 10^5^, and 10.0 × 10^5^ cells/dish). (C) Quantification of cells per microwell under various cell concentrations. (D) Diameter measurements of HDP cell spheroids over the culture period, with statistical significance indicated by *** (*P* < 0.001).

Next, to culture HDP cells as spheroids, we used specially designed dish. To generate the uniformly sized spheroids and prevent them from clumping together, we put a mesh grid in the dish and coated it with HGC to create an ULA environment. This setup helped the cells form 3D spheroids on the mesh (Fig. 2A). We seeded HDP cells at different concentrations: 2.0 × 10^5^, 5.0 × 10^5^, and 10.0 × 10^5^ cells per dish. Depending on the concentration, approximately 22, 40, or 100 cells entered each grid section (Fig. 2C). More cells entered the grid at higher concentrations. Within a day, these cells formed spheroids (Fig. 2B). The size of these spheroids also increased with the number of cells (76.0 ± 5.0, 87.2 ± 4.5, and 120.9 ± 4.6 μm for 2.0, 5.0, and 10.0 × 10^5^ cells/dish, respectively). Over a week, we did not observe any significant change in the size of the spheroids (Fig. 2D). It is important to note that in living organisms, dermal papillae at the base of hair follicles typically range from about 100 to 200 μm in size [7]. Therefore, we chose a cell seeding density of 10.0 × 10^5^ cells/dish as the optimal concentration for HDP cell spheroids in our subsequent experiments. It was observed that most spheroids maintained a live state at this density.

### Manufacturing and analysis of hair growth HDP cell spheroids

**Fig. 3.**
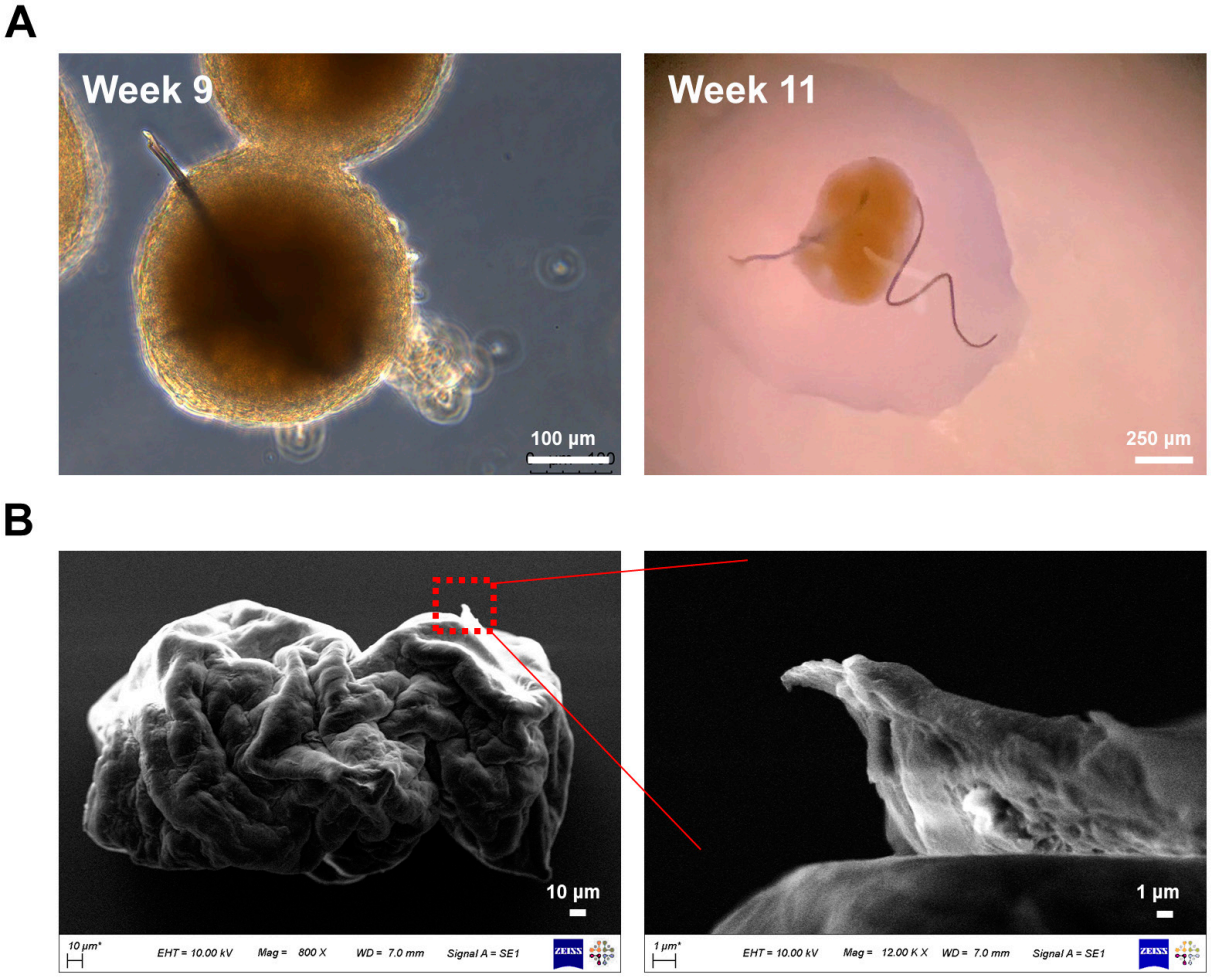
Formation of hair shaft-like structures by HDP cell spheroids cultured in mesh-integrated HGC-coated dishes. (A) Optical images at 9 and 11 weeks and (B) SEM images of HDP cell spheroids cultured for 7 weeks, showing the emergence of hair shaft-like structures.

To verify the hair-forming capabilities of HDP cell spheroids cultured in HGC-coated dish, they were maintained for an extended period. By 11 weeks, hair-like structures began to emerge from the spheroids, as depicted in Fig. 3A. These structures continued to grow longer over time. SEM analysis confirmed the presence of these protrusions from the cell clusters (Fig. 3B). Cells surrounding the perimeter of these structures, where they connected to the clusters, were also observed. Notably, some spheroids developed multiple hair-like structures (Fig. S2).

**Fig. 4.**
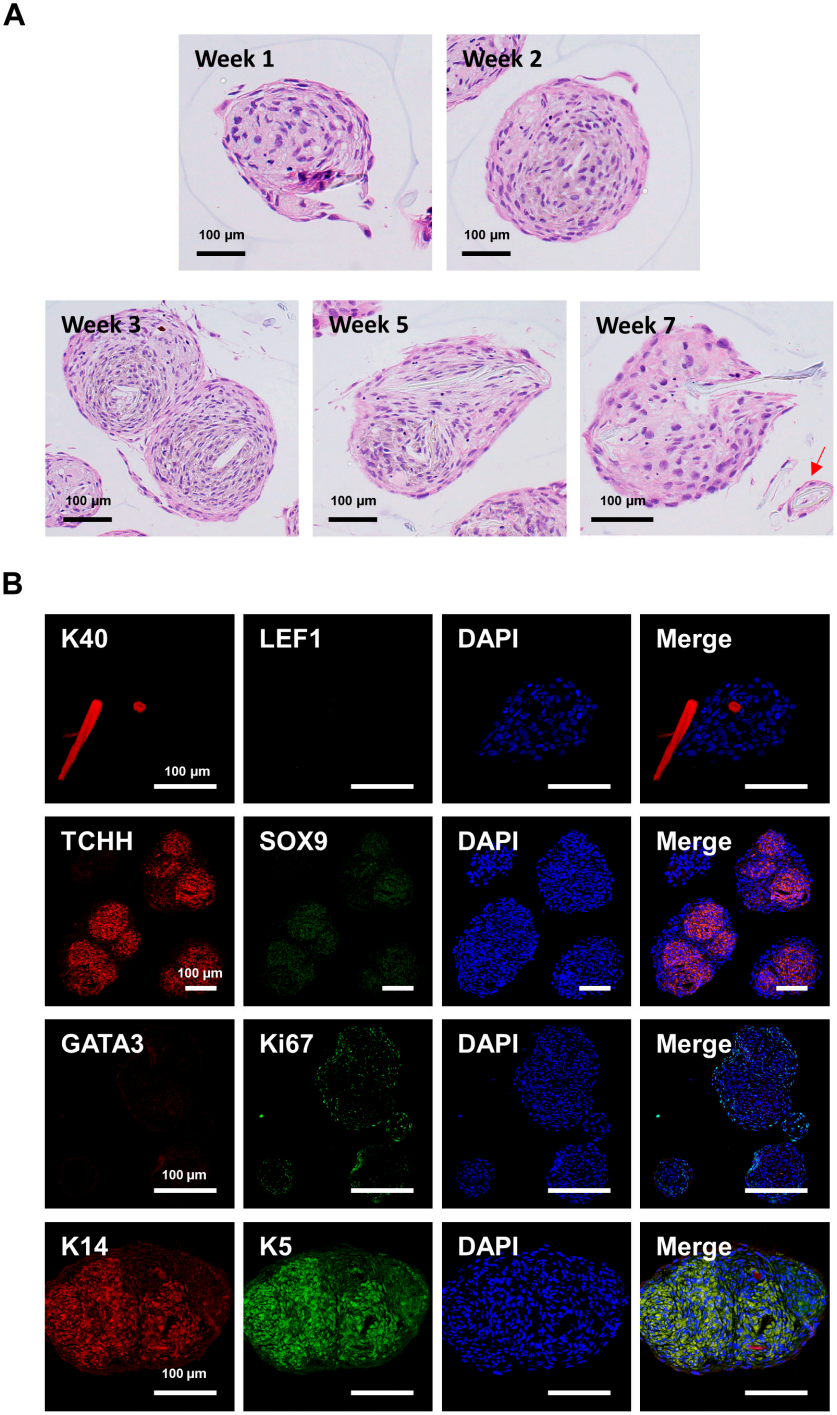
Characterization of HDP cell spheroids during hair shaft-like structure formation. (A) H&E staining of HDP cell spheroids cultured for 7 weeks, depicting the compact structure and progressive growth of hair shaft-like structures. The arrow points to the cross-section of a hair shaft-like structure. (B) Representative immunostaining images of HDP cell spheroids at week 7, showing positive staining for SOX9, Ki67, and K5 in green (Alexa Fluor 488); K40 and TCHH expression in red (Alexa Fluor 555), and DAPI staining for nuclei in blue.

For a closer look at the internal structure of spheroid with hair-like structures, we stained cross-sections of the spheroids with H&E at various intervals (Fig. 4A). Starting from the second week, the internal configuration began to change, differentiating from the first week, and started to resemble hair roots. By the 5 weeks, the development of hair-like structure within the HDP cell spheroids became distinctly visible. At 7 weeks, hair-like structures were seen emerging from the spheroids. The cross-section of hair-like structures at 7 weeks revealed these structures with hollow cores, akin to the medulla found in thick hair.

The results of the immunostaining are particularly revealing (Fig. 4B). The hair-like structures expressing K40 and K14 suggest that they are indeed analogous to real hair, as these keratins are key components of hair in humans. However, the absence of LEF1, a protein involved in hair follicle development, indicates that these structures, while similar, might not fully replicate all aspects of natural hair formation. In the HDP cell spheroids, the presence of TCHH, GATA3, K5, and K14, and the absence of SOX9, point toward a mature hair follicle-like phenotype. SOX9’s absence is particularly noteworthy as it is typically involved in hair follicle development and stem cell maintenance. This suggests that the cell clusters may be more differentiated and closer to a mature state than initial stem cell-like stage. The Ki67 staining results also provide valuable insights. The positive staining in the outer cells of the spheroids indicates active cell proliferation, a crucial aspect of hair follicle growth. The lack of Ki67 in the central cells could suggest that cells in the core of spheroid may undergo progression of differentiation into a more mature form.

### The response of HDP cell spheroids to minoxidil treatment

**Fig. 5.**
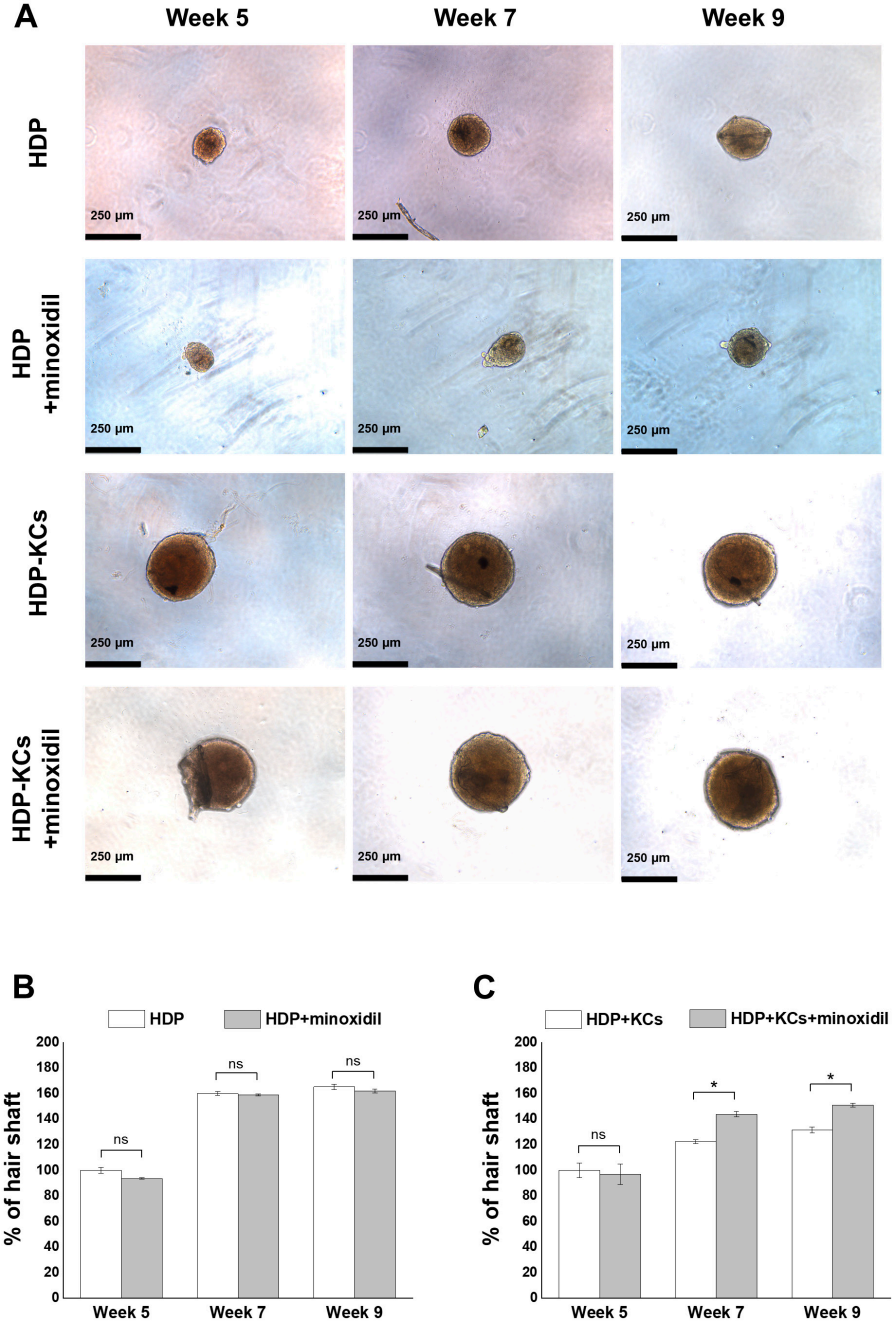
Effect of minoxidil on hair shaft-like structure formation in 3D mono-culture HDP cell spheroids and coculture HDP-KCs spheroids. (A) Representative phase-contrast images of HDP cell spheroids and HDP-KCs spheroids treated with and without 10 μM minoxidil for 9 weeks, displaying hair shaft sprouting. (B and C) Quantification of hair shaft-like structures sprouting in (B) HDP cell spheroids and (C) HDP-KCs spheroids in response to minoxidil treatment at weeks 5, 7, and 9 (*: *P* < 0.05; ns: no significance).

In light of these insights into the formation and characteristics of hair-like structures from HDP cell spheroids, we extended our study to assess their potential as a model for screening hair growth agents. We chose minoxidil for this purpose, given its status as a well-known and effective hair growth drug. We used spheroids cultured for 5 weeks for these tests, aligning with the timeline we observed for the onset of hair growth (Fig. 5A).

Interestingly, the response to minoxidil varied depending on the composition of the spheroids. In groups where the spheroids consisted solely of HDP cells, there was no noticeable effect on hair growth from the minoxidil treatment (Fig. 5B). This suggests that while these cells are critical for hair formation, they might not be the direct targets of minoxidil’s action. However, the result changed when HDP cells were combined with keratinocytes to form the spheroids. In these mixed-cell groups, the minoxidil-treated spheroids exhibited more than a 20% increase in hair growth compared to the untreated ones at 7 and 9 weeks (Fig. 5C). This result underscores the importance of cellular interactions in hair growth and the specific action mechanism of minoxidil.

These findings highlight the effectiveness of our spheroid production system from a new perspective. The system proves to be immensely useful not only in creating and studying the behavior of HDP cell spheroids but also in evaluating the potential of various hair growth agents, like minoxidil. By enabling precise control over the cellular composition and environment of the spheroids, our system offers a versatile platform for dissecting the complexities of hair growth and the efficacy of different treatments. This approach could significantly advance our understanding and development of more targeted, effective hair loss therapies.

### The potential in hair transplantation alternatives

**Fig. 6.**
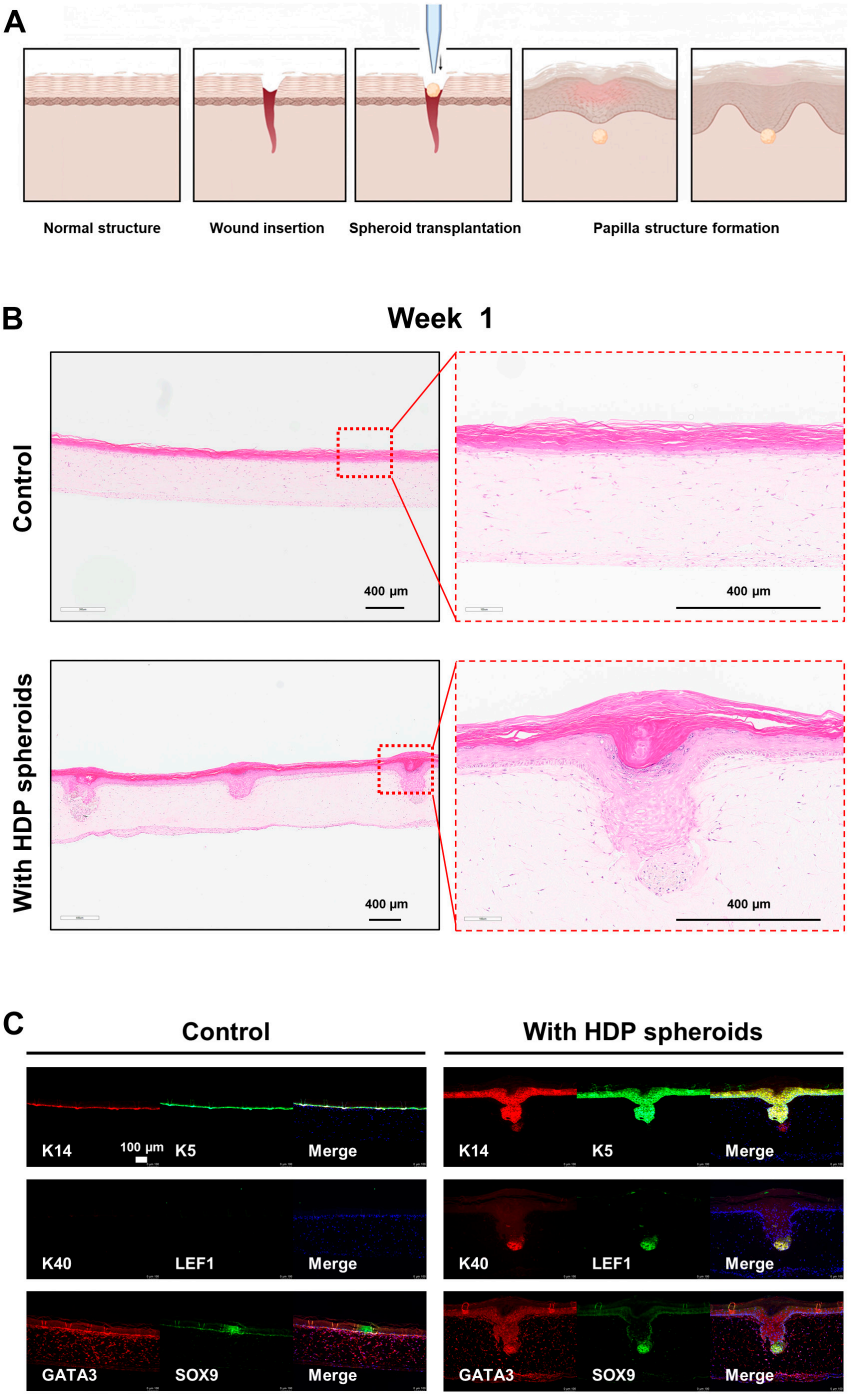
EpiDerm Full-Thickness (EpiDermFT) skin model incorporating HDP cell spheroids formed hair follicle papillary-like structure and hair shafts-like structure in vitro. (A) Schematic illustration of creating artificial skin with papillary-like structures by inducing a wound in EpiDermFT and incorporating HDP cell spheroids into the site. (B) Histological sections of spheroid-containing EpiDermFT tissue cultured for 1 week. (C) Representative immunostaining images of spheroid-containing EpiDermFT tissue cultured for 1 week, showing positive K14 and K5 staining for the basal epidermal layer in green (Alexa Fluor 488), DAPI staining for nuclei in blue, and positive staining for hair follicle development markers K40, LEF1, GATA3, and SOX9 in HDP cell spheroids in red (Alexa Fluor 555).

In our exploration of the applications of HDP cell spheroids, we conducted an experiment to assess their potential as a substitute for hair transplantation. This involved transplanting these spheroids into artificial skin. After making a precise incision in the artificial skin, the dermal papilla cell aggregates were carefully inserted (Fig. 6A). Figure 6B presents a control sample of normal artificial skin, which is a highly differentiated and multilayered model replicating human dermis and epidermis. This artificial skin features a well-organized structure, including basal, spinous, granular, and keratinized epidermal layers, similar to natural tissue. Remarkably, in the samples with transplanted HDP cell spheroids, we observed a thicker development of the basal layer compared to the control. Moreover, unique papilla structures that bridge the dermis and epidermis were formed, which were absent in the control group. These transplanted aggregates were also visible as protrusions extending from the dermis to the epidermis.

The results from the immunohistochemical analysis are crucial in understanding the biological implications of these findings (Fig. 6C). The presence of K14 and K5 in the epidermal layers of both control and transplanted samples indicates a healthy and typical epidermal differentiation. The exclusive expression of K40 and LEF1 in the dermal papilla cell aggregates located in the dermal protrusions suggests that these aggregates are actively involved in the formation of hair-like structures. LEF1, in particular, is a key regulator in hair follicle development, underscoring the functional potential of these aggregates in hair-like structure formation. Furthermore, GATA3 expression in both the dermis and epidermal layers of both groups is indicative of healthy epidermal differentiation and maturation. The presence of SOX9 exclusively in the HDP cell spheroids aligns with its role in hair follicle development and stem cell maintenance, reinforcing the notion that these spheroids are critical in initiating hair-like structure development.

**Fig. 7.**
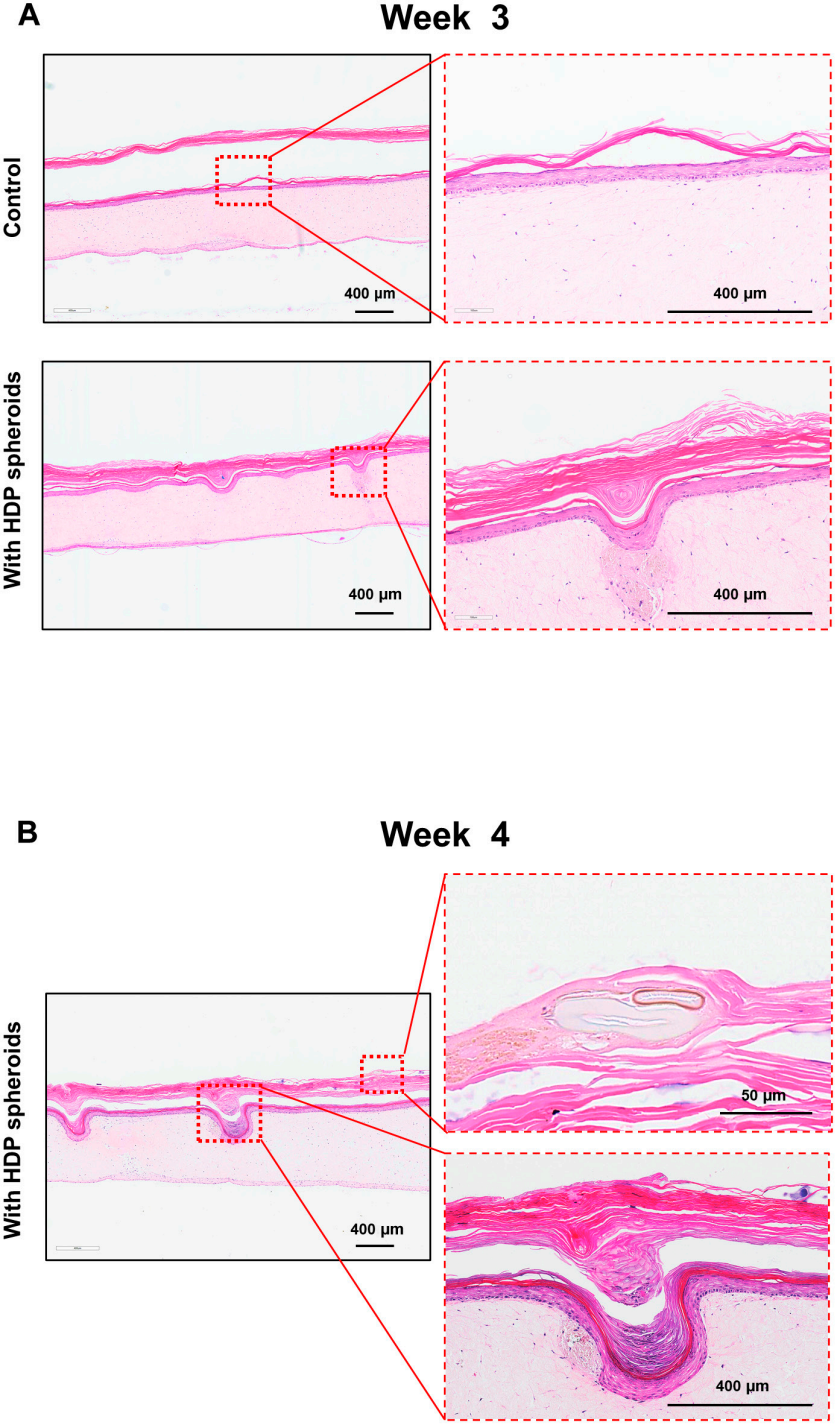
Histological sections of spheroid-containing EpiDermFT tissue cultured for (A) 3 weeks and (B) 4 weeks.

To examine the longevity of this structure, we extended the culture period beyond 3 weeks. In the control group, there was a complete separation of the epidermal and keratin layers after 3 weeks, and the thickness of the basal layer significantly diminished (Fig. 7A). In contrast, the samples with transplanted HDP cell spheroids maintained their papilla structures similar to their state in week 1. Furthermore, the basal layer, spinous layer, granular layer, and keratinized epidermal layer were well preserved. Most notably, by week 4, hair-like structures were observed in the keratin layer of the group with transplanted aggregates (Fig. 7B). These structures, as depicted in Fig. 4A, possessed a hollow interior, further signifying their resemblance to natural hair. This experiment not only proposed HDP cell spheroid transplantation as an alternative to traditional hair transplantation techniques but also demonstrates the feasibility of using advanced artificial skin models as a realistic platform for hair regeneration research.

## Discussion

This study successfully demonstrated the preparation and application of HDP cell spheroids in 3D cultures, highlighting the potential of HGC-coated environments for forming hair-like structures. This achievement stems from the unique properties of HGC, notably its ultralow cell adhesion.

In this study, HDP cell spheroids were able to form hair-like structures within a 3D culture system, tackling a major challenge in the field: replicating complex physiological processes similar to natural hair growth. Our findings indicate that these spheroids can emulate aspects of natural hair, as evidenced by their expression of keratins K40 and K14 (Fig. 4B). While we did not achieve fully functional hair follicles, the formation of hair-like structures in vitro using only dermal papilla cells is a noteworthy advancement, not previously reported in the literature. Hair formation in vitro has typically involved cocultures of keratinocytes and papilla cells [2,7,21–25]. Generating hair-like structures using only papilla cells represents a significant advance and presents a new model for understanding hair growth mechanisms. This success is likely due to the ULA environment created by HGC, which played a critical role in the unique behavior and differentiation of the papilla cells. Additionally, our research showed the sustainability of hair growth over extended periods, with observations extending beyond 7 weeks (Fig. 4A). This consistent growth of hair-like structures was facilitated by a specially designed culture dish that allowed for the uniform creation and maintenance of the spheroids. The ability to sustain these structures over long periods signals the potential for more extensive studies and applications in the future.

Our study demonstrates the potential of 3D cell culture systems as effective platforms for testing the efficacy of hair growth agents. Our study was evaluated minoxidil on our 3D HDP cell spheroids, assessing their potential as an alternative to animal models in drug testing. The differential response to minoxidil between spheroids composed solely of papilla cells and those cocultured with keratinocytes is particularly revealing. The results suggest that 3D cell culture systems can be fine-tuned to better understand the mechanisms of action of hair growth drugs. These results have significant implications for developing animal-free drug evaluation systems. It underscores the complexity of hair follicle biology and the importance of cellular interactions in hair growth [16,23–27]. The enhanced response to minoxidil in the presence of keratinocytes suggests that both cell types play crucial roles in the hair growth process and should be included in drug testing models for hair loss treatments.

Our findings also extend to the application of HDP cell spheroids in artificial skin, leading to the formation of hair-like and papilla structures, crucial for skin functionality. The enhanced functionality of this improved artificial skin was evident in several aspects. For instance, we observed that the keratin layer did not separate, indicating enhanced structural integrity and resilience (Fig. 7A). These improvements in the artificial skin model are not just academically significant. In addition, the closer resemblance of the artificial skin to real human skin increases its utility as a model for various applications. One of the most promising applications of our improved artificial skin is as an alternative model for cosmetic and pharmaceutical evaluations [28–30]. The ability to test products on skin that closely mimics human skin can lead to more accurate and relevant results. This is especially important in an era where ethical concerns and regulatory constraints regarding animal testing are increasing [2,11]. Furthermore, the advancements in our artificial skin model hold potential for clinical applications, particularly in treating extensive skin damage. The ability to cultivate and integrate functional papilla structures within the artificial skin enhances its potential for use in skin grafting procedures, offering a promising technology for patients suffering from severe skin injuries or diseases. This could significantly improve the outcomes of such treatments in terms of functionality and aesthetic results.

Our study not only demonstrates the feasibility of using 3D cell culture system for hair growth research but also represents advancements in artificial skin technology. These contributions will be useful in a wide range of applications, including dermatological research, cosmetic and pharmaceutical testing, and potentially clinical settings for skin repair and regeneration.

## Ethics Approval and Consent to Participate

Not applicable

## Data Availability

For data requests, please contact the authors.
