# Supplementary material for: Three-Dimensional Cultured Human Dermal Papilla Cells in HGC-Coated Environments Enhance Hair Regeneration and Artificial Skin Integration
Source: Biomater Res. 2024 Apr 12;28:0018. doi: 10.34133/bmr.0018 (PMC12237074; doi:10.34133/bmr.0018)
Supplement: Supplementary 1 — Figs. S1 to S2 [file bmr.0018.f1.docx]

Supplementary Information

**Three-Dimensional Cultured Human Dermal Papilla Cells in HGC-Coated Environments Enhance Hair Regeneration and Artificial Skin Integration**

Thuy Trang Truong^†,‡,1^, Heejin Park^§,1^, Kyoung Hwan Park^‡^, Jin Jung Song^§^, Byoung-Seok Lee^§^, Kang Moo Huh ^‡,*^, and Sun-Woong Kang^†,||,*^

^†^Research Group for Biomimetic Advanced Technology, Korea Institute of Toxicology, Daejeon 34114, Republic of Korea

^‡^Department of Polymer Science and Engineering, Chungnam National University, Daejeon 34134, Republic of Korea

^§^Department of Toxicological Evaluation and Research, Korea Institute of Toxicology, Daejeon 34114, Republic of Korea

^||^Human and Environmental Toxicology School, University of Science and Technology, Daejeon 34114, Republic of Korea

^1)^These authors contributed equally to this work as first author.

^*)^These authors contributed equally to this work as corresponding author.

***Corresponding authors**

Sun-Woong Kang, Ph.D.

E-mail address: swkang@kitox.re.kr

Kang Moo Huh, Prof.

E-mail address: khuh@cnu.ac.kr

**Supplementary Figure 1**


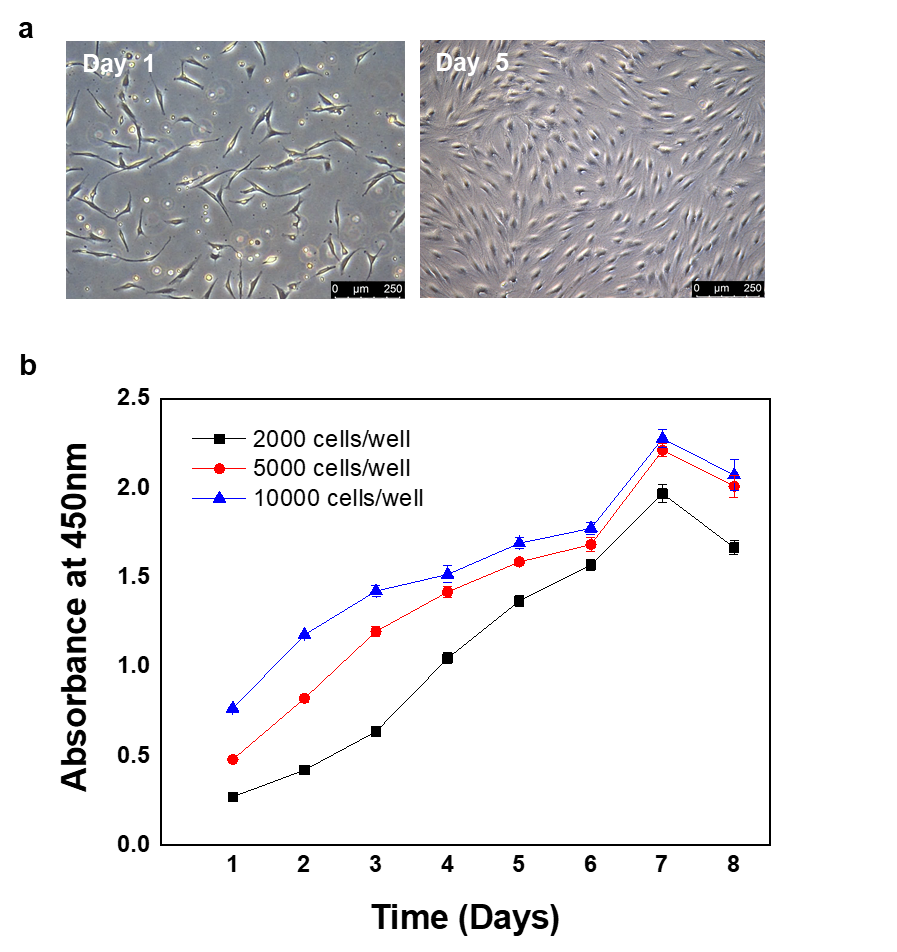


Supplementary Fig. 1 Morphology and Growth Rate of HDP Cells in Two-Dimensional (2D) Culture. (a) Optical images showing the morphology of HDP cells cultured in 2D culture dishes. (b) Graphical representation of cell proliferation assessed using the Cell Counting Kit-8 (CCK-8).

**Supplementary Figure 2**


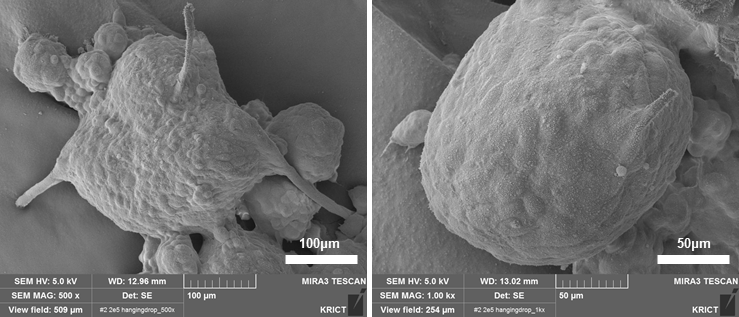


Supplementary Fig. 2 Scanning Electron Microscopy (SEM) Images of HDP Cell Spheroids. SEM images of HDP cell spheroids cultured for 7 weeks. These images illustrate the emergence of hair shaft-like structures from the spheroids.
